# A Case of Broken Local Anesthetic Needle in the Pterygomandibular Space; Diagnostic Approaches and Surgical Management

**DOI:** 10.3390/diagnostics13193050

**Published:** 2023-09-25

**Authors:** Ziad Malkawi, Alaa Alayeh, Abedalaziz Alshawa, Ola Shaban, Omar Al Saraireh, Hashem Malkawi, Hamzah Babkair, Ismail Abdouh, Najla Dar-Odeh

**Affiliations:** 1 School of Dentistry, The University of Jordan, Amman 11942, Jordan; z.malkawi@ju.edu.jo (Z.M.); alaaayea@yahoo.com (A.A.); abd8191786@ju.edu.jo (A.A.); ala8191784@ju.edu.jo (O.S.); 2 King Hussein Cancer Center, Amman 11941, Jordan; oa.11038@khcc.jo; 3 Royal College of Surgeons in Ireland Medical University in Bahrain, Manama 15503, Bahrain; hashemmalkawi@yahoo.com; 4 College of Dentistry, Taibah University, Al Madinah Al Munawara 43353, Saudi Arabia; hbabkair@taibahu.edu.sa (H.B.); iabdouh@taibahu.edu.sa (I.A.)

**Keywords:** broken needle, CBCT, dental anesthesia, inferior alveolar nerve block, pterygomandibular space

## Abstract

Needle fracture during dental local anesthetic injections is a rare but significant, potentially serious complication. Various approaches for the location and removal of broken needles have been described; however, there are several difficulties and concerns related to the potential complications and critical anatomic challenges peculiar to the head and neck region. In this case, we describe the diagnostic approaches utilized in locating a broken needle that migrated in the pterygomandibular space following gag reflex, and sudden head movement of a middle-aged male patient. A meticulous diagnostic approach was employed to locate the needle utilizing CBCT scan, CT scan with contrast, and C-arm X-ray machine. The needle was successfully retrieved using an angled hemostat inserted through an oral incision, guided by a C-arm X-ray machine and ENT endoscopic instruments. While careful planning could prevent many complications that may arise during oral surgical procedures, inadvertent events leading to serious complications should be addressed using the appropriate and timely diagnostic techniques pre-and intra-operatively.

**Figure 1:**
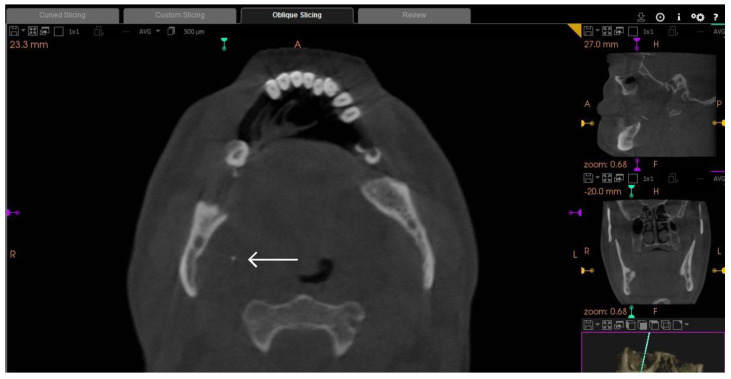
Axial section of CBCT taken immediately after needle fracture showing the anterior portion of the needle (arrow). The needle was directed obliquely vertical to the point of insertion and medial to the medial pterygoid muscle. Fracture of local anesthetic needles during injection has been described, particularly during the inferior alveolar nerve block, which is considered the most common local anesthetic procedure to be used intraorally, and to be complicated by needle fracture [1]. The incidence is reported to be 1 in 14 million inferior alveolar nerve blocks [2]. Upon the occurrence of this unfortunate event, the surgeon is faced by a number of options: to leave in situ or, more favorably, to remove as soon as possible to avoid the anticipated complications of pain, trismus, infection, and damage to important structures of blood vessels and nerves [3]. Needles broken during inferior alveolar nerve block may migrate in the pterygomandibular space where the mandibular nerve and inferior alveolar blood vessels are located, which presents a surgical challenge because of the interplay of complicated access, the close anatomic relationship to important structures, and the small diameter of the anesthetic needle fragment This report describes a case of a local anesthetic needle that was broken during injection procedure. It also describes diagnostic methods utilized and the surgical method that was followed to retrieve the broken needle. A 50-year-old, medically fit non-smoking male presented for the extraction of his lower-right third molar which had been affected by severe caries. Upon initiation of the inferior alveolar nerve block anesthesia using a disposable 23 mm 30-gauge dental needle, the patient developed a gag reflex and suddenly moved his head. The surgeon attempted to remove the needle; however, the needle fractured and the broken needle was out of sight. The surgeon estimated that the broken needle disappeared completely in the pterygomandibular space medial to the ramus. A decision was made to take a CBCT scan (Cone beam computed tomography) immediately to visualize the broken segment. (Figure 1).

**Figure 2:**
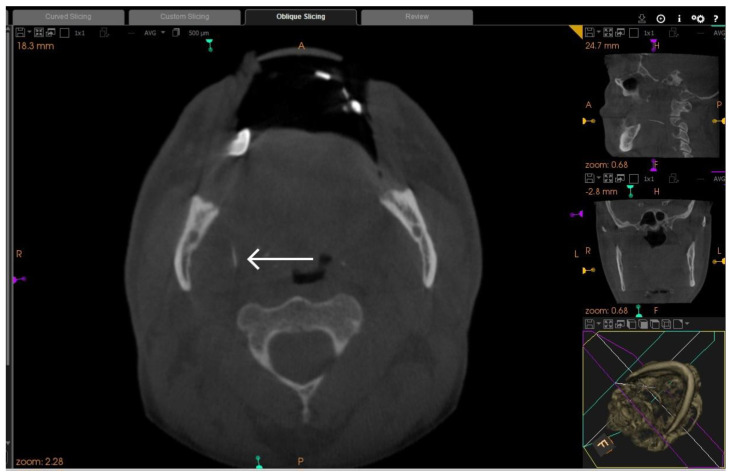
Axial section of CBCT taken five days later showing the anterior part of fractured needle (arrow) in a more posterior position compared to first CBCT image. The needle moved to a deeper location, more inferiorly and medially to the medial pterygoid muscle approaching the junction with lateral pharyngeal space, which necessitated removal under GA. An appointment was given to arrange for removal of the needle under LA and IV sedation after five days. Preoperative CBCT scan and CT-scan with contrast were requested to evaluate the needle and the soft tissue structures around the fractured part (Figure 2 and Figure 3).

**Figure 3:**
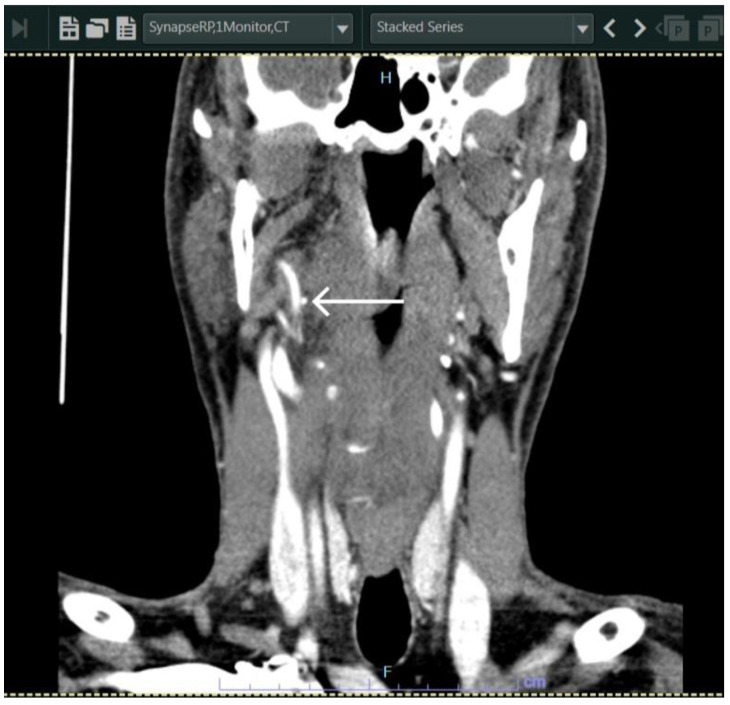
Coronal section of CT with contrast taken immediately preoperatively, showing the fractured needle in close proximity (arrow) to a large vessel. The needle was identified in the most medial part of the right medial pterygoid muscle, close to the plane between the medial pterygoid muscle and the superior pharyngeal constrictor muscle. Consent was obtained from the patient to operate under general anesthesia using either a transoral or transcervical approach. The patient was placed in the supine position, general anesthesia was induced, and the oral cavity was prepared and draped in. Oral intubation was used and the endotracheal tube was inserted and placed on the other side (left side of the oral cavity) so as not to interfere with the field of interest.

**Figure 4:**
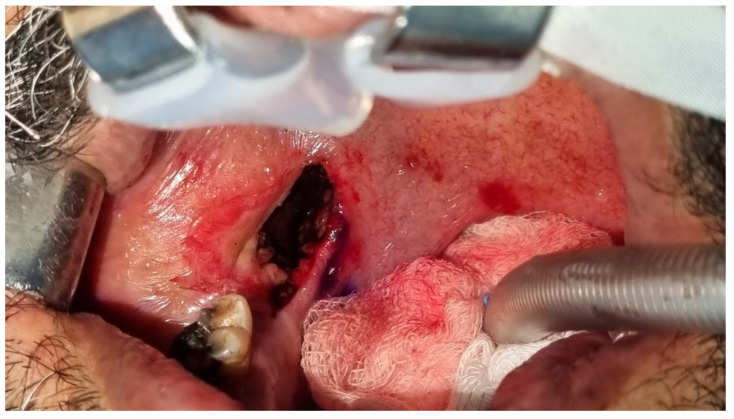
A transoral approach was employed. A throat pack was inserted, and the mouth was opened wide using a Boyle Davis mouth gag, adult size with 75 × 25 blade. Infiltration of lidocaine 2% with 1:100,000 epinephrine (Lignospan, Septodont) was administered at the surgical site for additional hemostasis, and an incision was made (Figure 4). A 2 cm mucosal incision was made through the mucosa and submucosal tissues just medial to pterygomandibular raphe until the fascia of the medial pterygoid was identified. The plane between the medial pterygoid and superior constrictor muscles was accessed via blunt dissection along the raphe, creating a tunnel-like pathway. Hemostasis was achieved via electrocautery and topical application of adrenaline-soaked gauze throughout the procedure. An angled 20 cm hemostat was used for retrieval (Figure 5 and Figure 6).

**Figure 5:**
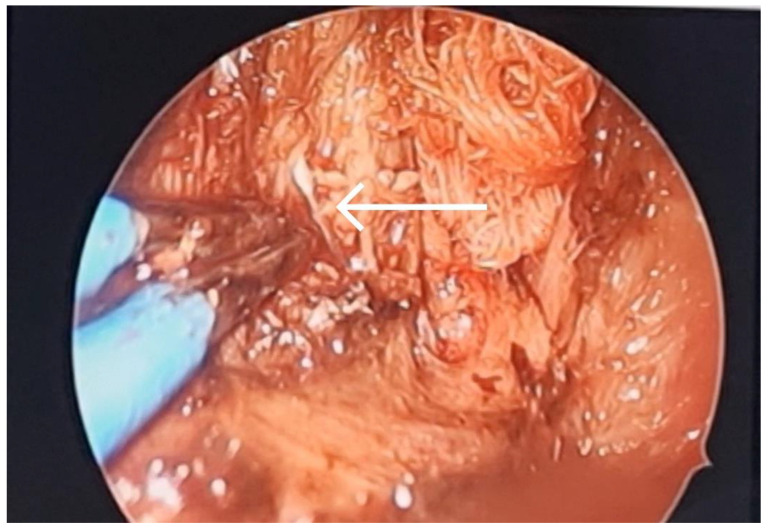
A curved hemostat was inserted through the oral incision for retrieval of the needle (arrow) guided via C-arm X-ray machine with the assistance of ENT endoscopic instruments (Medtronics sinus endoscope with zero-degree angle) for dissection of the medial mandibular area.

**Figure 6:**
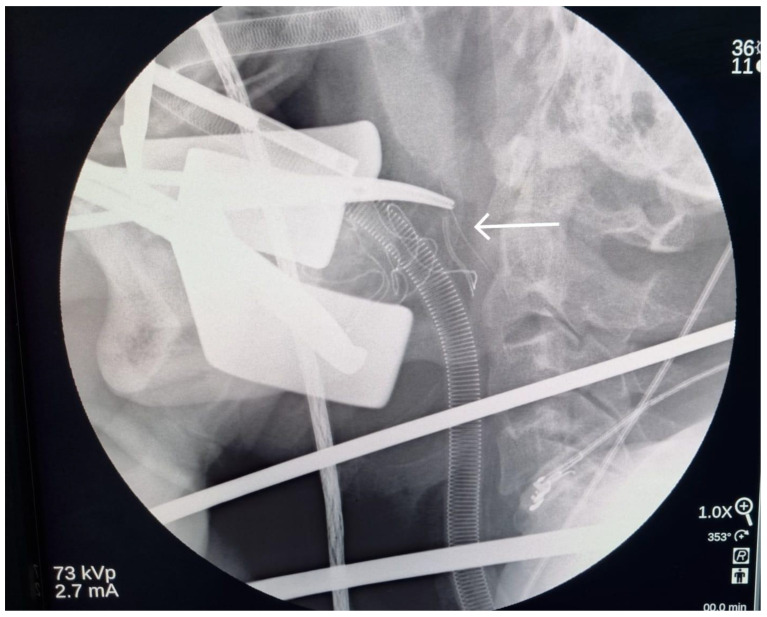
Multiple X-ray images (lateral neck view) were taken to insure the adequate depth of dissection. When the adequate depth was achieved, the angled hemostat was placed just posterior to the needle on lateral neck view, and a postero-anterior neck X-ray was taken to identify the fractured needle location on the vertical plan. It was just lateral to the hemostat (arrow), which was placed in medial pterygoid-superior constrictor plane. The hemostat was slightly withdrawn and directed to dissect carefully through the medial pterygoid muscle until a part of the fractured needle was located with the assistance of the endoscope.

**Figure 7:**
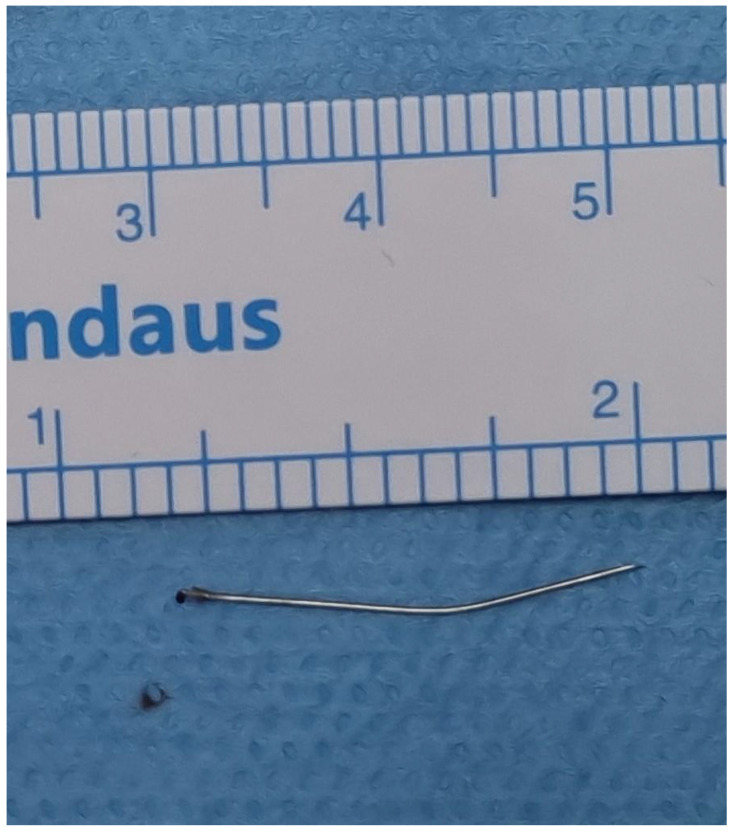
Broken needle after removal. Careful manipulation and maneuvering were performed to grasp and remove the fractured needle carefully (Figure 7). The area was inspected for any additional fragments or injury, and no other abnormalities were noted. Adequate suctioning and irrigation were performed to ensure the removal of any debris or foreign material from the surgical site upon confirming the complete needle retrieval. Hemostasis was ensured, the surgical site was irrigated with sterile saline, and the wound was inspected for any signs of bleeding. The submucosal tissues were re-approximated with resorbable sutures (VICRYL Plus 3 0 polyglactin 910, ETHICON), followed by closure of the mucosa with interrupted resorbable sutures to enhance wound healing [4]. Postoperatively, the patient was monitored in the recovery room before being transferred to the ward in a stable condition. Instructions were given for postoperative care, including pain management, oral hygiene, and follow-up appointments. In the weekly follow-up visit, sufficient healing was observed and no complications were reported. Treatment outcomes of this case were favorable due to utilizing appropriate diagnostic approaches and management technique, which took into consideration the characteristic anatomic factors relevant to this area. During the procedure of inferior alveolar block, the needle passes through the pterygomandibular space, which contains the inferior alveolar bundle (nerve, artery and vein), the lingual nerve, the nerve to mylohyoid, the sphenomandibular ligament, and fascia. While injury to any of these structures could ensue with the anticipated morbidities of neurological deficits and bleeding, another possibility could be related to migration of the needle to neighboring more critical spaces. In this case, an inappropriate needle type was used which was an important factor predisposing to needle fracture. The appropriate type is a long needle approximately 32 mm with a gauge of 25 or 27. Most clinicians recommend removal of broken needles due to the anticipated psychological and infectious complications. Furthermore, needle migration is a potential consequence that can endanger important structures in the neighboring areas [5]. Needles broken in the pterygomandibular space may migrate into the lateral pharyngeal space, where the internal maxillary artery, the ascending pharyngeal artery, and pterygoid venous plexus are located [5]. After fracture of the needle the surgeon may be faced with two scenarios: the needle tip may be visible which entails the use of a curved hemostat or equivalent to aid retrieval. Another scenario could be encountered when the needle disappears within the mucosa; herein, no effort should be made to retrieve it manually, as palpation of the soft tissue may lead to further migration. The practitioner should instruct the patient to avoid jaw movements to minimize needle migration, and immediate referral should be made to the nearest oral and maxillofacial surgery unit for emergency assessment and management. Diagnostic approaches to locate the broken needle prior to surgical removal are of the utmost importance. It is particularly important to maintain a fixed position of the patient’s jaw during pre- and intraoperative scanning. This was achieved during the management of our patient, and during the operation, the patient’s mouth was kept open maximally using a mouth gag to obtain a fixed and well-defined open position for the accurate identification of the needle. Plain radiography may be used initially in the form of panoramic radiograph or plain radiographic films taken perpendicular to each other. A disadvantage of plain radiography is the lack of accuracy in estimating the needle position in relation to neighboring structures. Digital radiography represents another option, due to its various advantages. In addition to its cost effectiveness, which makes it suitable for use in low-resource settings, it has the ability to manipulate radiographic views by zooming in and changing the contrast [6]. However, a more accurate technique such as CBCT is a more favorable approach preoperatively, due to its applicability and accuracy in the maxillofacial region [7,8]. A recent case report described the use of a 3D technique based on CBCT, whereby a 3D-printed surgical guide was used to locate the needle [9]. In this case, we used CBCT initially and later, contrast-enhanced CT was used. Enomoto et al. (2009) emphasized the importance of using contrast-enhanced CT in evaluating the positional relationship of foreign bodies trapped in the parapharyngeal space and the nearby large vessels, namely jugular vein, maxillary, and carotid arteries [10]. During the operation, several techniques for identifying the broken needle in pterygomandibular space have been described. Thompson et al. (2003) employed a stereotactic technique that utilized two venipuncture needles, which were sequentially placed until they meet at the broken needle portion radiographically [11]. Blunt dissection was then performed along one of these needles until the tip of the broken fragment was grasped. However, intraoperative plain radiography may be time-consuming and challenging to perform. Additionally, it can be difficult to evaluate small positional changes on plain radiographs. C-arm digital fluoroscopy may facilitate rapid imaging without disturbance of the reference needles [12]. Nonetheless, the two-dimensional images of fluoroscopy represent a limitation similar to plain radiography, which may hinder the provision of a precise position of the needle fragment. Therefore, the operator would have to examine images in different directions to accurately locate the needle. Intraoperative ultrasonography has also been described in locating foreign bodies in the neck. It has the advantage of minimizing radiation exposure; however, as with plain radiography and fluoroscopy, ultrasonography does not provide the exact position and the small area of the oral cavity is not compatible with the size of the ultrasound device [13]. Metal detectors were described as a means for locating fractured needles [14], based on their ability to determine the change in the induction of a search coil, and producing tones that change upon approximating a metallic object. A metal detector can differentiate between different metals in the orofacial region, such as dental amalgam and dental needles. However, metal detectors have a relatively large size and they are not readily available. On the other hand, new technologies have been developed recently to identify lost metallic sharps in the form of radiofrequency-based navigation systems [15]. In the past, some clinicians recommended the use of an electromagnet to locate and remove broken instruments; however, this method is not applicable for the needles used recently since they are made of stainless steel devoid of iron [16]. In recent times, surgical navigation systems can be employed accurately to locate broken needle fragments [17]. These systems employ a computer workstation, a display system, a tracking system, and a set of surgical instruments. Various means of tracking are available for locating needles such as the use of infrared, optical tracking, and electromagnetism. The infrared model may be helpful in locating instruments that are to be tracked. It is based upon releasing invisible light from an overhead infrared unit which is then reflected to an overhead camera by specialized reflective equipment, thereby locating the required objects. The use of optics-based navigation systems entails a modification of the operation theater setup to reduce optical interferences and maintain a line of sight during tracking. The line-of-sight problem is not encountered when using the electromagnet-based navigation systems; however, metallic or electromagnetic instruments should be kept at a distance during tracking because of their ability to interfere with the navigation system [15]. A nasal endoscope was used to retrieve the broken needle in our patient. Transoral endoscopic retrieval of broken needles was mentioned by Lehmann in 2020 in a report of four cases of broken needles dislodged in the infratemporal space during local anesthesia, and all were successfully retrieved [18]. In summary, this case report highlights the importance of employing the appropriate local anesthetic technique to avoid needle fracture in the critical clinical scenario of inferior alveolar block anesthesia, especially in patients anticipated to be uncooperative. It is also important to adopt a well-planned line of management for anesthetic needle fracture in the maxillofacial region, including the utilization of appropriate diagnostic imaging techniques and therapeutic measures. The use of the endoscope, image guidance, and the transoral approach can be carefully employed to promote the success of the retrieval technique.

## Data Availability

Data available on request due to privacy restrictions.
